# Seminal Plasma Modulates miRNA Expression by Sow Genital Tract Lining Explants

**DOI:** 10.3390/biom10060933

**Published:** 2020-06-19

**Authors:** Isabel Barranco, Lorena Padilla, Cristina A. Martinez, Manuel Alvarez-Rodriguez, Inmaculada Parrilla, Xiomara Lucas, Graça Ferreira-Dias, Marc Yeste, Heriberto Rodriguez-Martinez, Jordi Roca

**Affiliations:** 1 Department of Medicine and Animal Surgery, Faculty of Veterinary Medicine, University of Murcia, E-30100 Murcia, Spain; isabel.barranco@udg.edu (I.B.); lorenaconcepcion.padilla@um.es (L.P.); parrilla@um.es (I.P.); xiolucas@um.es (X.L.); 2 Biotechnology of Animal and Human Reproduction (TechnoSperm), Department of Biology, Institute of Food and Agricultural Technology, University of Girona, E-17003 Girona, Spain; marc.yeste@udg.edu; 3 Department of Biomedical & Clinical Sciences (BKV), BKH/Obstetrics & Gynaecology, Faculty of Medicine and Health Sciences, Linköping University, SE-58185 Linköping, Sweden; cristina.martinez-serrano@liu.se (C.A.M.); manuel.alvarez-rodriguez@liu.se (M.A.-R.); 4 CIISA—Centre for Interdisciplinary Research in Animal Health, Faculty of Veterinary Medicine, University of Lisbon, 1300-477 Lisbon, Portugal; gmlfdias@fmv.ulisboa.pt

**Keywords:** explants, female genital tract, immune response, miRNAs, mucosal tissue, seminal plasma, transcriptome

## Abstract

The seminal plasma (SP) modulates the female reproductive immune environment after mating, and microRNAs (miRNAs) could participate in the process. Considering that the boar ejaculate is built by fractions differing in SP-composition, this study evaluated whether exposure of mucosal explants of the sow internal genital tract (uterus, utero-tubal junction and isthmus) to different SP-fractions changed the profile of explant-secreted miRNAs. Mucosal explants retrieved from oestrus sows (*n* = 3) were in vitro exposed to: Medium 199 (M199, Control) or M199 supplemented (1:40 *v*/*v*) with SP from the sperm-rich fraction (SRF), the post-SRF or the entire recomposed ejaculate, for 16 h. After, the explants were cultured in M199 for 24 h to finally collect the media for miRNA analyses using GeneChip miRNA 4.0 Array (Affymetrix). Fifteen differentially expressed (False Discovery Rate (FDR) < 0.05 and Fold-change ≥ 2) miRNAs (11 down- versus 4 up-regulated) were identified (the most in the media of uterine explants incubated with SP from post-SRF). Bioinformatics analysis identified that predicted target genes of dysregulated miRNAs, mainly miR-34b, miR-205, miR-4776-3p and miR-574-5p, were involved in functions and pathways related to immune response. In conclusion, SP is able to elicit changes in the miRNAs profile secreted by female genital tract, ultimately depending SP-composition.

## 1. Introduction

Mounting evidence suggests that seminal plasma (SP), the complex fluid secreted mainly by the male accessory sexual glands, is not a mere sperm carrier, but it also plays a crucial role in other reproductive physiology events [1,2,3]. In effect, previous studies indicate that the cross-talk between SP-components and the female reproductive system induces molecular and cellular changes, conditioning the success of ovulation, fertilization, embryo implantation, pregnancy and even the health of the offspring [1,4,5,6]. Particular suggestions have been made of the immunoregulatory properties of SP as potentially responsible for that effect, since this fluid contributes to the establishment of a tolerogenic immune environment in the female genital tract, which is required for pregnancy success [4,7,8,9].

In pigs, in vivo studies have revealed that SP is able to modulate the maternal environment, interacting with the female reproductive tract and modifying the expression of certain genes, mainly those that are immune response-related [10,11]. While these findings increase our understanding on how SP is involved in the regulation of the female environment in this species, its impact on the gene-expression profile may not be enough to explain effects on protein translation [12]. In this context, one should wonder about the role of microRNAs (miRNAs), since these small endogenous non-coding RNAs (~22 nucleotides) are known to be crucial post-transcriptional regulators of gene expression, promoting degradation of target mRNAs and/or modulating their translation [13,14]. In addition, accumulating evidence supports that one miRNA targets multiple mRNAs, which suggests that a given miRNA may exert its influence across a wide variety of gene expression networks [15].

The role of miRNAs as regulators of several biological processes, including reproductive functions, has attracted significant attention in the last years [16,17,18,19]. In the female reproductive tract, several miRNAs have been identified as responsible for regulating cellular pathways essential for their correct functions [18,20], also serving as biomarkers of pathological processes [16,21]. Accordingly, the last studies in this realm have reported that certain miRNAs expressed by female reproductive tissues, mainly the endometrium, are involved in the regulation of genes involved in pregnancy [22]. In the pig, it has been demonstrated that miRNAs expressed by the endometrium could play a crucial role for successful embryo implantation and placentation [23,24,25]. Given the involvement of miRNAs in the modulation of several immunological pathways [26,27,28], it is reasonable to think that miRNAs expressed by the female genital tract could modulate proper maternal immune environment required for successful pregnancy [17,28,29]. The miRNAs are not only limited to an intracellular location, since they can be secreted, mainly via extracellular vesicles, to the extracellular environment and then taken up by different cells, modulating its gene transcription [30]. Studies performed in mice and humans demonstrated that endometrial miRNAs could act as regulators of embryo-maternal cross-talk [31,32,33], which suggests that these secreted miRNAs could also modulate the maternal intrauterine environment. Therefore, studies focusing on the evaluation of changes in the secretion of immune-regulatory miRNAs following exposure of female genital tissues to SP are warranted, since, as mentioned previously, miRNAs account for the control of gene expression networks. Moreover, the boar ejaculates in well-defined fractions with clear temporal delivery and specific composition [34], ruling from carrying the sperm vanguard [34] to the bulk of major proteins, eliciting inflammatory uterine responses [5,35]. An obvious question is whether the different fractions, or the entire ejaculate, induce differential responses from specific compartments of the female mucosa, focusing on miRNAs secreted to the culture medium.

Against this background, the present study evaluated whether in vitro exposure of mucosal explants of the internal genital tract of oestrus sows (uterus, utero-tubal junction (UTJ) and isthmus) to SP from different fractions (i.e., the sperm-rich fraction (SRF), the post-SRF) or a composite (recomposed entire ejaculate (EE)), changed the profile of secreted miRNAs. 

## 2. Materials and Methods

### 2.1. Experimental Design

Mucosal explants (*n* = 72) were retrieved from the two side-segments (right and left) of the uterus, UTJ and isthmus (four explants per each side-segment) of three sow (24 explants per sow) and were individually cultured in Medium 199 (M199) at 38 °C in 5% CO_2_ in air in a humidified incubator (CO₂ Incubator C60, Labotect Labor-Technik-Göttingen GmbH, Göttingen, Germany) under continuous gentle shaking (Orbit™ P2 Digital Shaker, Labnet International Inc., Edison, NJ, USA) for 60 min. Thereafter, the four explants from the same segment/side/sow were separately subjected to the following treatments (as described below): (1) M199 (Control), and (2) M199 supplemented (1:40 *v*/*v*) with pooled SP from (2a) the SRF, (2b) the post-SRF or (2c) the recomposed EE. After a 16 h incubation period, the media were harvested and replaced with 1 mL of fresh M199 and incubated under the same conditions for a further 24 h, to finally collect the medium (mixing the medium of the left and right sides from the same segment/treatment/sow (2 mL)), which was frozen in liquid nitrogen and stored at −80 °C (Ultra Low Freezer; Haier Inc., Qingdao, China) until being examined for miRNA analysis profiling. 

### 2.2. Ethics Statement

All procedures were performed according to the European Directive 2010/63/EU EEC for animal experiments and approved by the Bioethics Committee of Murcia University (research code: 639/2012).

### 2.3. Reagents and Media

The chemicals used in the experiments were of analytical grade. Unless otherwise stated, all reagents were acquired from Merck KgaA (Darmstadt, Germany) and the media were prepared under sterile conditions within a laminar flow hood (MicroH, Telstar, Terrasa, Spain). 

### 2.4. Boars, Ejaculates and Seminal Plasma

Ejaculates used in the experiment were collected from five healthy, mature (2–3 years old) and fertile boars of different crossbreeds (Landrace × Large-White) belonging to a center for artificial insemination (AI, AIM Iberica, Calasparra, Murcia, Spain). One ejaculate per each boar was collected in two separate fractions using the gloved-hand method: the SRF and the post-SRF. A representative proportion of each fraction was thereafter mixed to mimic a recomposed EE. The ejaculates used in the experiment fulfilled the standards of quantity and quality for commercial AI-doses (˃200 × 10^6^ spermatozoa/mL, 70% of them motile and 75% of them morphologically normal).

Right after manual semen collection, samples from each of the two ejaculate fractions and the recomposed EE were double centrifuged (1500× *g* for 10 min at room temperature (RT, Rotofix 32A; Hettich Centrifuge UK, Newport Pagnell, Buckinghamshire, England, UK)), in order to obtain the SP. The resultant SP samples were examined microscopically (Eclipse E400; Nikon, Tokyo, Japan) to ensure they were sperm-free. Thereafter, the SP-samples were pooled per fraction and EE, stored in conical tubes (15 mL) and shipped in insulated containers with dry ice to the Laboratory, where they were stored at −80 °C (Ultra-Low Freezer). 

### 2.5. Sows, Tissue Collection and Explant Preparation

Endometrial (mid-cornual endometrium) and endosalpingeal (UTJ and isthmus segments) explants were obtained from three multiparous (3–6 parity) healthy crossbred (Landrace × Large White) sows from a commercial farm (PORCISAN, Las Palas, Murcia, Spain). The sows were checked for signs of oestrus (twice per day), tested by experienced staff by applying manual back-pressure on the standing sows during snout-to-snout contact with a mature boar. Once sows showed a standing oestrus reflex, animals were subjected to surgery in a specific surgical room located on-farm. Sows were sedated by administration of azaperone (Stresnil, Dr. Esteve S.A, Barcelona, Spain; 2 mg/kg body weight, intramuscular) and general anesthesia was induced using sodium thiopental (Tiobarbital, B. Braun VetCare S.A, Barcelona, Spain; 7 mg/kg body weight, intravenous). Anesthesia was maintained with 3% to 5% isoflurane (IsoFlo, Abbott Laboratories S.A., Madrid, Spain). Sows were subjected to mid-ventral laparotomy, and after exposure of the reproductive tract, ovaries were morphologically examined, confirming the presence of pre-ovulatory 5–8 mm follicles. The surgical procedure followed the protocol described by Martinez et al. [11]. Then, samples of endometrium (middle portion of each uterine horn) and endosalpinx (from left and right UTJ and isthmus) were excised and then further dissected, caring for the lining epithelium to have a minimal portion of the underlying layers, using sterile scissors. Thereafter, each explant was cut with scalpel blades into small similar size pieces (weight: 30–40 mg; hereinafter referred to as mucosal explants). Laparotomy was layer-sutured using Coated VICRYL^®^ (Polyglactin 910, Ethicon Inc., Somerville, NJ, USA), and sows returned to recovering crates after intramuscular administration of a long-acting amoxicillin suspension (15 mg/kg body weight). 

### 2.6. Explant Culture 

Under sterile conditions, within a laminar flow hood cabinet (MicroH, Telstar), explants (30–40 mg of tissue each) were each transferred individually into a well of a 24-well plate (Nunc™, Thermo Fisher Scientific, Waltham, MA, USA) with 1 mL of culture medium per well (M199), supplemented with 0.1% bovine serum albumin, 0.075 g/L penicillin G, 0.05 g/L streptomycin sulphate and 2.2 g/L NaHCO_3_ (pH 7.5 ± 0.1; 297 ± 2 mOsmol/kg). The explants were cultured for 60 min at 38 °C in a humidified atmosphere containing 5% CO_2_ (CO₂ Incubator C60) with gentle shaking (Orbit™ P2 Digital Shaker) prior to each treatment, as described in the Experimental Design Section. The media were collected for storage and an aliquot was indirectly analyzed for cell viability of the explant through measurement of lactate dehydrogenase (LDH) activity using an automated analyzer (AU 600, Olympus, Minneapolis, MN, USA). The LDH activity (mU/mg of tissue cultured, expressed as mean ± SEM) was in uterus: 13.52 ± 0.17 mU/mg, in UTJ: 11.45 ± 0.2 mU/mg, and in isthmus: 26.32 ± 0.98 mU/mg. 

### 2.7. RNA Isolation

A commercially available kit specifically designed to isolate small RNAs in fluids (miRNeasy serum/plasma kit, Qiagen, Hilden, Germany) was used to extract RNA from our samples. In brief, 200 μL of each sample was thawed on ice, mixed (1:1; v:v) with QIAzol Lysis Reagent, incubated (5 min at RT) and then 200 μL of chloroform was added. The resulting sample was mixed by agitation (15 s at RT), incubated (3 min at RT) and centrifuged (12,000× *g* at 4 °C for 15 min). Two phases were obtained and 400 μL of the upper phase was extracted and mixed with 600 μL of 100% ethanol. Then, the resultant mixture (700 μL) was placed into a 2 mL tube with RNeasy MinElute spin column and centrifuged (8000× *g* at RT for 15 s) to allow the RNA to attach onto the column membrane, discarding the flow-through. The process was repeated with the rest of the sample with the same column. Thereafter, the column was successively washed with RWT buffer (700 μL), RPE buffer (500 μL) and 80% ethanol (500 μL), centrifuging (8000× *g* at RT for 15 s (RWT and RPE) or for 2 min (ethanol)) and discarding the flow-through each time. Lastly, the column was centrifuged (15,000× *g* at RT for 6 min), removing the flow-through, and the RNA was obtained by adding 14 μL nuclease-free water onto the column membrane and centrifuging (15,000× *g* and RT for 1 min). Total RNA concentration and purity were assessed by NanoDrop^®^ 1000 (Thermo Fisher Scientific) and quality was determined with microfluidic electrophoresis (2100 Bioanalyzer, Agilent Technologies, Santa Clara, CA, USA). Only samples with high RNA concentration were stored at −80 °C for its further analysis, because miRNA profiling required a minimum concentration of 16.25 ng/μL. RNA integrity number (RIN) values were 2.36 ± 0.08 (mean ± SEM) and the A_260_/A_280_ ratio was ˃1.7 in all cases. These low RIN values have been demonstrated to exert negligible or no effect on miRNA profiling analysis due to the robust stability of miRNAs [36,37,38].

### 2.8. miRNA Microarray Analysis

Affymetrix^®^ GeneChip miRNA 4.0 Arrays (Thermo Fisher Scientific) were utilized to perform the miRNA expression profiling. An Affymetrix^®^ FlashTag^TM^ Biotin HSR RNA Labelling Kit (Thermo Fisher Scientific) was used to create Biotin-labeled RNA. In brief, isolated RNA (130 ng) were subjected to poly-A tail incorporation to the 3’-end, and then a biotin-labeled 3DNA molecule was linked to the 3′-end by a DNA ligase. The resultant biotin-labeled RNA samples were hybridized to the GeneChip miRNA 4.0 arrays in an Affymetrix^®^ Oven 455 (Thermo Fisher Scientific) (18 h at 48 °C with 60 rpm rotation). After hybridization, miRNA 4.0 arrays were washed, stained and scanned by the Affymetrix^®^ GeneChip^TM^ Hybridization Wash and Stain Kit (Thermo Fisher Scientific), Affymetrix^®^ Fluidic Station 450 (Thermo Fisher Scientific) and Affymetrix^®^ GeneChip Scanner 3000 7G (Thermo Fisher Scientific), respectively. 

### 2.9. Bioinformatics of Microarray Data

CEL-files of raw data were produced with the Affymetrix^®^ GeneChip Command Console Software (version 4.0, Thermo Fisher Scientific), and further analyzed using the Affymetrix^®^ Transcriptome Analysis Console software (version 4.0, Thermo Fisher Scientific), after normalizing the intensity by the robust multi-array average. Three comparisons were performed per explant, as follows: SP from the SRF versus control, SP from post-SRF versus control and SP from recomposed EE versus control. *p*-values were adjusted through the Benjamini–Hochberg False Discovery Rate (FDR). miRNAs that fulfilled the criteria (*p*-value ˂ 0.05 or FDR < 0.1 or 0.05 and ≥2.0-fold-change (FC) ≤ −2.0) were extracted as significantly up- or down-regulated miRNAs. Principal Component Analysis (PCA) and hierarchical clustering were performed to check grouping of samples based on dysregulated miRNAs using Affymetrix^®^ Transcriptome Analysis Console software v4.0. The raw datasets were deposited at Gene Expression Omnibus (GEO) (https://www.ncbi.nlm.nih.gov/geo/) with the identifier (GSE149079).

### 2.10. Target Gene Prediction and Functional Analysis

Target genes of dysregulated miRNAs were predicted using miRDB (http://mirdb.org) [39]. Gene Ontology (GO) enrichment and pathway analysis of predicted-targeted, dysregulated miRNA genes were performed with DAVID (database for annotation, visualization and integrated discovery) and KEGG (Kyoto Encyclopedia of Genes and Genomes database) databases. The network of biological function and pathways based on the GO and KEGG databases was investigated using Cytoscape Software v3.0.0 (http://www.cytoscape.org/) application ClueGO v2.0.3. 

## 3. Results

Three statistical criteria were applied during screening of differentially expressed (up/down) miRNAs in the culture media harvested from the three different explants of the female genital tract examined (uterus, UTJ and isthmus), following exposure to different treatments (M199 supplemented with SP from SRF, post-SRF and EE) in relation to controls (i.e., those expressed in the media from the same explants only cultured with M199). Regardless of the statistical criteria applied, miRNAs were firstly selected using a FC cut-off of ≤−2 and ≥2. The first criterion consisted of the conventional statistical significance level set at *p*-value < 0.05 (criterion 1). In the following increasingly restrictive criteria, FDR was used and considered to be significant when the *q*-value threshold was <0.1 (criterion 2) or <0.05 (criterion 3). 

**Figure 1:**
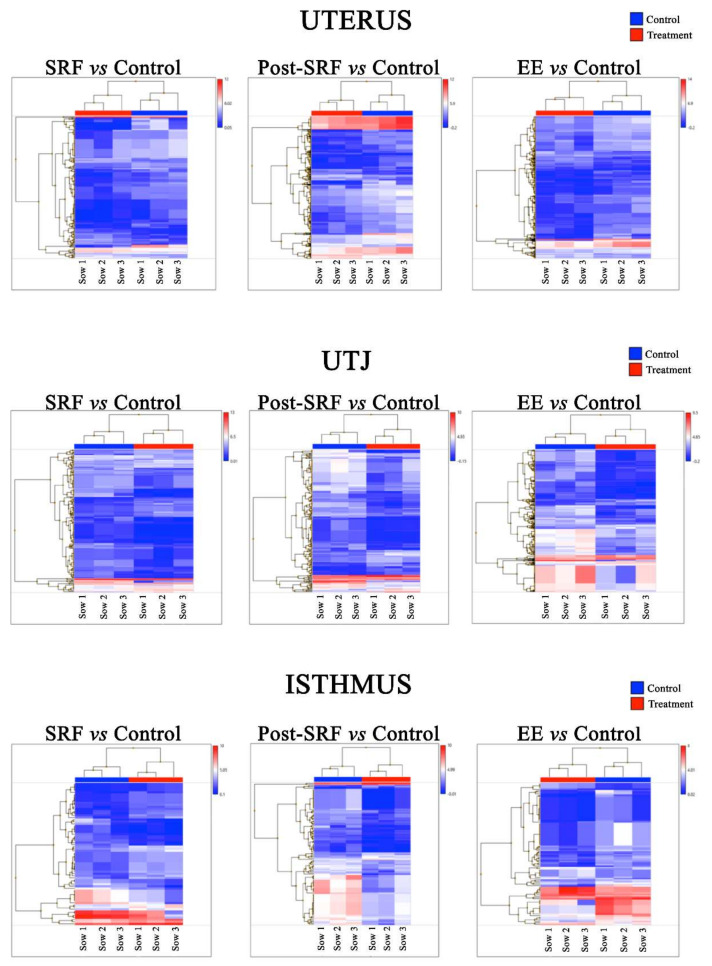
Hierarchical clustering of the differentially expressed microRNAs (miRNAs) (*p*-value ˂ 0.05 and ≥2.0-Fold Change (FC) or ≤−2.0) in media of each cultured explant (uterus, utero-tubal junction (UTJ) and isthmus) exposed to different treatment (Medium 199 (M199) supplemented with seminal plasma from different ejaculate fractions (sperm-rich fraction (SRF) and post-SRF) and from the entire recomposed ejaculate (EE)) compared with its control (C, M199 alone), showed a clear distinction between control and treatments in the three sows. The color scale indicates the relative expression of miRNAs: red shows higher expression and blue lower expression. Each row represents one miRNA and each column represents a tissue sample.

When criterion 1 was used, the hierarchical cluster analysis applied to the miRNA expression profiles of each explant resulted in a clear separation between treatments and controls, showing that the three sows had the same pattern (Figure 1). Three components were obtained in the PCA based on identified miRNAs, explaining up to 75.8% of the variance (PC1: 54.4%, PC2: 13.7% and PC3: 7.7%; Supplementary Figure S1).

The number of differentially expressed miRNAs (up/downregulated) in the medium from each explant and treatment applying the three statistical criteria are shown in Supplementary Table S1. A total of 1302 differentially expressed miRNAs were found applying criterion 1 (*p*-value < 0.05), and a lower amount of differentially expressed miRNAs was identified using criterion 2 (FDR < 0.1: 55 miRNAs), or criterion 3 (FDR < 0.05: 41 miRNAs). Regardless of the statistical criteria used, the number of downregulated miRNAs was higher than the number of miRNAs upregulated in the culture medium from all explants and treatments compared with their respective controls. Similarly, differential expression of most miRNAs was higher in utero- and UTJ- than in isthmus-media (Supplementary Table S1).

**Figure 2:**
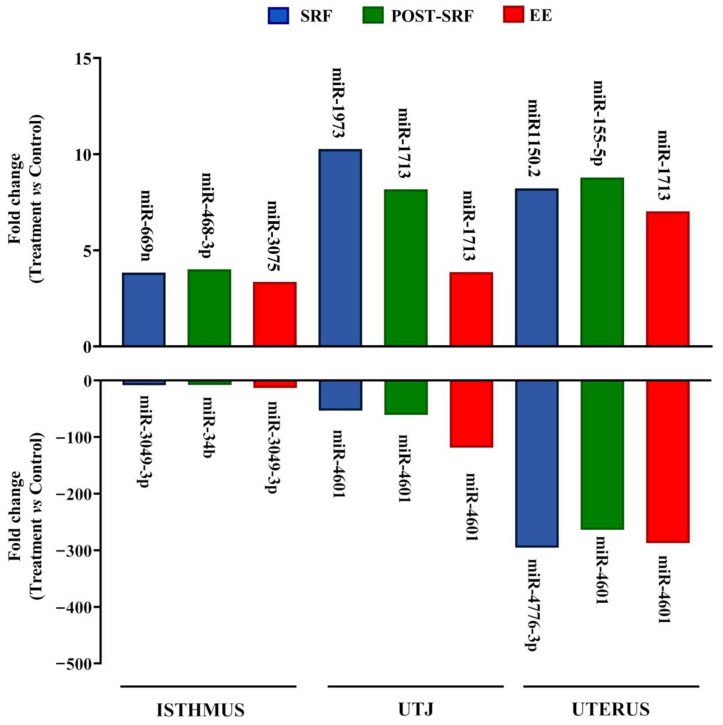
Differentially expressed microRNAs (miRNAs, *p*-value < 0.05) in culture media from explants (uterus, utero-tubal junction (UTJ) and isthmus) exposed to different treatment (Medium 199 (M199) supplemented with seminal plasma from different ejaculate fractions (sperm-rich fraction (SRF) and post-SRF) and the recomposed ejaculate (EE)) compared to controls (M199 alone). Bar chart showing the miRNAs down- and up-regulated in each explant and treatment with the highest and lowest fold change value.

Differentially expressed miRNAs identified after applying the first criterion (*p*-value < 0.05) are listed in Supplementary Table S2 (including miRNA name (ID), *p*-value, FC and whether they were up- or down-regulated). Here, the number of overlapping differentially expressed miRNAs in the medium of each explant is summarized in Venn diagrams (Supplementary Figure S2). The list of differentially expressed miRNAs that are common in the different treatments is provided per explant in Supplementary Table S3. Likewise, Venn diagrams depict the overlapping differentially expressed miRNAs for each treatment (Supplementary Figure S3). The minimum and maximum FC values in each explant-medium and treatment for the differentially down- and up-regulated miRNAs are shown in Figure 2. 

A more restrictive analysis was carried out applying criterion 2 (FDR ˂ 0.1) reducing the number of differentially expressed miRNAs (Supplementary Table S4, including miRNA name (ID), FDR *q*-value, FC and its direction (up- or down-regulated)). Applying this statistical cut-off, none of the previous differentially expressed miRNA was identified in the medium from cultured isthmus. A small number of upregulated miRNAs was found in all uterine media and only in the medium of UTJ exposed to SP from SRF (Supplementary Figure S4). miRNAs differentially expressed overlapping in each treatment are shown in Supplementary Figure S5. 

**Figure 3:**
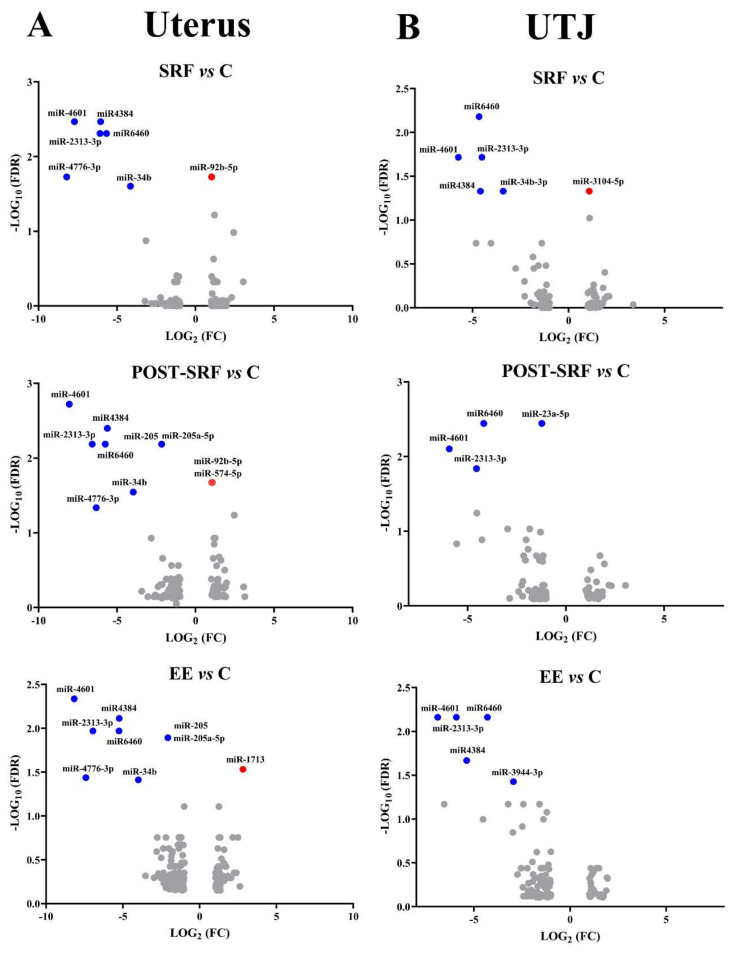
Volcano plots showing microRNAs (miRNAs) differentially expressed (*p*-value < 0.05 and ≥2.0-Fold Change (FC) or ≤−2.0-FC) in the medium retrieved from (**A**) uterus and (**B**) utero-tubal junction (UTJ) mucosal explants exposed to different treatments: Medium 199 (M199) supplemented with seminal plasma from different ejaculate fractions (sperm-rich fraction (SRF) and post-SRF) and from the entire recomposed ejaculate (EE), compared with its control (C, M199 alone). Top left blue dots represent downregulated miRNAs with a False Discovery Rate (FDR) < 0.05 and FC > −2 and top right red dots represent upregulated miRNAs with an FDR < 0.05 and FC > 2.

**Figure 4:**
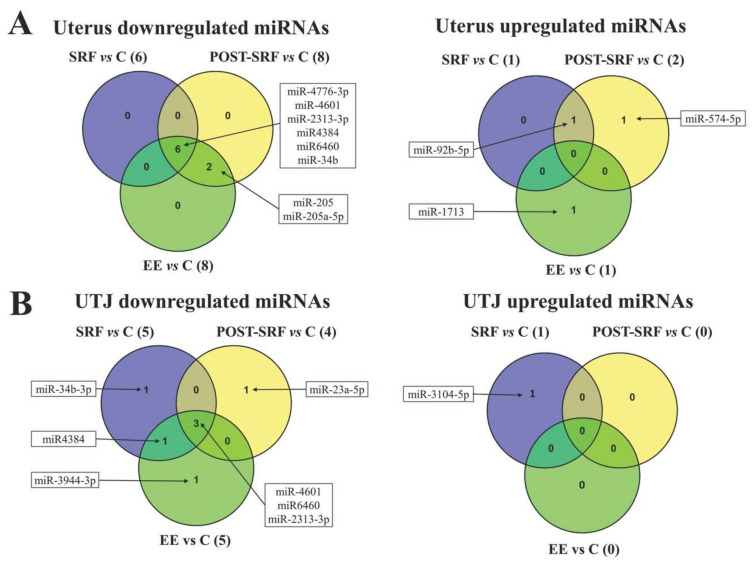
Venn diagram depicting the microRNAs (miRNAs) differentially regulated (False Discovery Rate < 0.05 and ≥2.0-Fold Change or ≤−2.0) and overlapped in the media of each incubated explant (uterus and utero-tubal junction (UTJ)) exposed to different treatment (Medium 199 (M199) supplemented with seminal plasma from different ejaculate fractions (sperm-rich fraction (SRF) and post-SRF) and the recomposed ejaculate (EE) compared with its control (C, M199 alone). (**A**) MiRNAs downregulated (left) and upregulated (right) in utero-culture medium. (**B**) MiRNAs downregulated (left) and upregulated (right) in UTJ-media.

**Figure 5:**
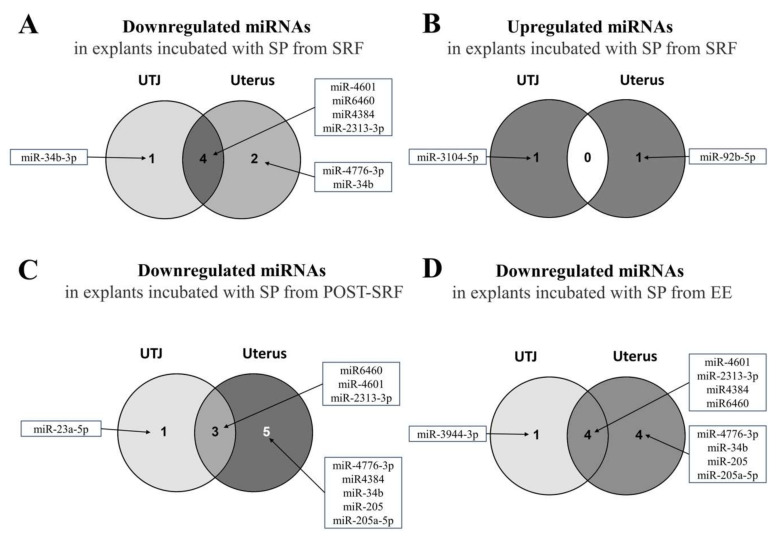
Venn diagrams showing the microRNAs (miRNAs) differentially regulated (False Discovery Rate < 0.05 and ≥2.0-Fold Change or ≤−2.0) in the media of mucosal explants and overlapped by each experimental treatment. The explants (uterus and utero-tubal junction (UTJ)) were cultured with medium 199 (M199) alone (Control) or supplemented with seminal plasma (SP) from different ejaculate fractions (sperm-rich fraction (SRF) and post-SRF) and from the recomposed ejaculate (EE). (**A**) MiRNAs downregulated in the media harvested from explants incubated with SP from SRF. (**B**) MiRNAs upregulated in the media from explants incubated with SP from SRF. (**C**) MiRNAs downregulated in the media from explants incubated with SP from post-SRF. (**D**) MiRNAs downregulated in the media from explants incubated with SP from EE.

Finally, the differentially expressed miRNAs still identifiable after applying the most stringent filtering (FDR < 0.05, criterion 3) are presented in Figure 3. The miRNAs that were overlapped between UTJ and uterus are shown in Figure 4, whereas Figure 5 displays those miRNAs that were overlapped between treatments, either in the UTJ or in the utero. Taking into account all differentially expressed miRNAs among treatments and explants, a total of 15 miRNAs were identified, namely miR6460, miR-4601, miR-2313-3p, miR4384, miR-34b-3p, miR-4776-3p, miR-34b, miR-205, miR-205a-5p, miR-23a-5p, miR-92b-5p, miR-3104-5p, miR-3944-3p, miR-574-5p and miR-1713. The analysis revealed that miR-4601, miR-2313-3p and miR6460 were the common downregulated miRNAs in UTJ- and utero-media, regardless of SP-source used. Similarly, miR4384, miR-34b and 4776-3p were the common downregulated miRNAs in media of utero-explants in all treatments. miR4384 was identified in culture media of UTJ exposed to SP from SRF or EE. The miR-205 and 205-5p were downregulated in uterine explant media incubated with SP from post-SRF or EE. Similarly, miR-92b-5p was upregulated in uterine media incubated with SP from SRF or post-SRF. By contrast, the specific miRNAs of each SP-source were: miR-34b-3p (down) and miR-3104-5p (up) in medium from UTJ incubated with SP from SRF, miR-23a-3p (down) and miR-574-5p (up) in media from UTJ and uterus respectively, incubated with SP from post-SRF and miR-3944-3p (down) and miR-1713 (up) in culture media of UTJ and uterus respectively, incubated with SP from EE. 

**Figure 6:**
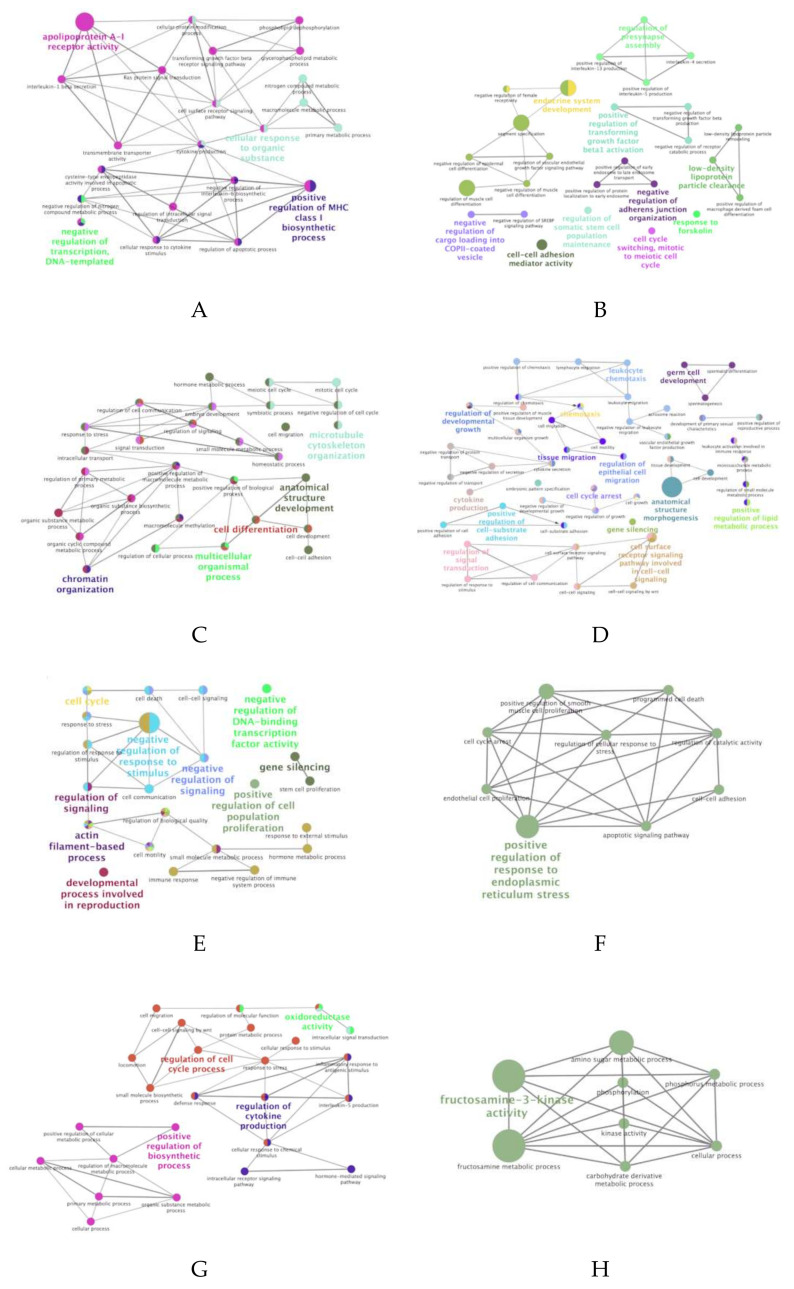
Schematic representation of biological processes associated with target genes of downregulated microRNAs (miRNAs) ((**A**): miR-23a-5p, (**B**): miR-34b, (**C**): miR-34b-3p, (**D**): miR-205, (**E**): miR-4776-3p, (**F**): miR-3944-3p) and upregulated ((**G**): miR-92b-5p, (**H**): miR-574-5p) identified in media of explants (uterus and/or utero-tubal junction) exposed to Medium 199 (M199) supplemented with seminal plasma from different ejaculate fractions (sperm-rich fraction (SRF) and post-SRF) and the recomposed ejaculate, compared with its control (M199 alone). Cytoscape v3.0.0 application ClueGO v2.0.3 was used to perform the analysis of overrepresented functional categories. The following databases were used: Gene Ontology (GO) subgroups biological process. Terms are functionally grouped based on shared genes (kappa score) and are shown in different colors. The degree of significance is indicated by the size of the nodes: the biggest nodes correspond to the highest significance. The name of the group is defined by the most significant term. The following ClueGO parameters were used: biological process database (BP; date: 28.03.2019), GO tree levels, 2–5 (first level = 0), minimum number of genes, 2, minimum percentage of genes, 2, GO term fusion, GO term connection restriction (kappa score), 0.4, GO term grouping, initial group size of 2 and 50% for group merge. The resulting network was modified, which means that some redundant and unnecessary terms were removed and the network was manually rearranged.

**Figure 7:**
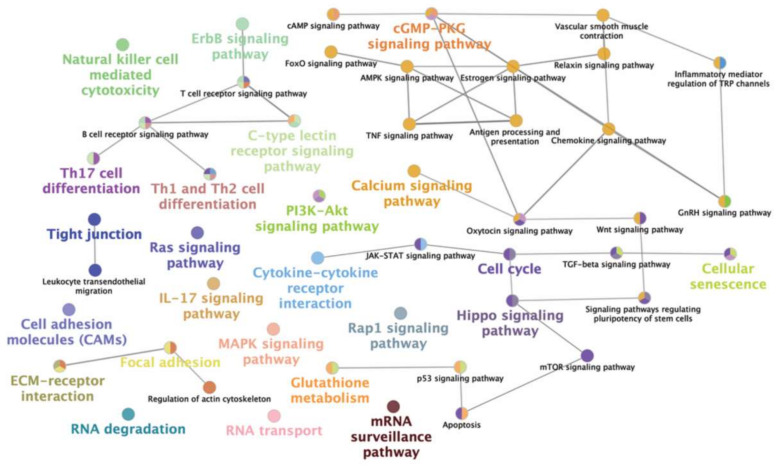
ClueGO network of the main pathways examined with the KEGG (Kyoto Encyclopedia of Genes and Genomes) database of predicted target-genes of differentially expressed microRNAs (miRNAs) (False Discovery Rate < 0.05 and ≥2.0-Fold Change or ≤−2.0) in culture media of explants (uterus and/or utero-tubal junction) exposed to Medium 199 (M199) supplemented with seminal plasma from different ejaculate fractions (sperm-rich fraction (SRF) and post-SRF) and the entire recomposed ejaculate (EE), compared with its control (M199 alone).

Among the 15 differentially expressed miRNAs (criterion 3), the predicted target genes for eight of them were found using the miRNA-target database (miRDB). Since the miRNA-target database has been developed for model organisms (mainly humans), the porcine sequences of those miRNAs described were compared to their human homologs, confirming their high homology (http://www.mirbase.org). To reduce the number of false positives, we listed only potential target genes with a score ≥0.9 [40]. Table 1 shows the 433 predicted target genes putatively regulated by our eight differentially expressed miRNAs. GO biological process analysis (ClueGO network of GO terms) based on predicted target-genes of dysregulated miRNAs showed terms mainly involved in biological processes, such as regulation of cytokine production, anatomical structure morphogenesis, regulation of response to stimulus, regulation of epithelial cell migration or cell differentiation, amongst others (Figure 6). ClueGO software (KEGG) revealed the implication of miRNA-target genes in several immune-related pathways, such as Th1 and Th2 cell differentiation, cytokine-cytokine receptor interaction, T cell receptor signaling pathway, TGF-beta signaling pathway and pathways involved in cellular processes, such as PI3K-Akt signaling pathway, focal adhesion, cell adhesion molecules, MAPK signaling pathway, Wnt signaling pathway, amongst others (Figure 7). A more exhaustive analysis evidenced that miR-34b, miR-205, miR-4776-3p and miR-574-5p were the main dysregulated miRNAs involved in reproduction and immune pathways (Table 2). All of these miRNAs were identified in culture media of explants incubated with SP from post-SRF (miR-34b, miR-205, miR-4776-3p and miR-574-5p) and some of them were also found in those incubated with SP from SRF (miR-34b and miR-4776-3p) and EE (miR-34b, miR-205 and miR-4776-3p). 

## 4. Discussion

There is extensive evidence supporting the concept that the SP modulates the female reproductive tract immune environment after mating, allowing the establishment of an adequate status of maternal immune tolerance at the peri-conception period, which is required for a subsequent successful pregnancy [4,8,9,41]. However, the specific mechanisms via which SP modulates this immune response still remain unknown. Schjenken et al. [28] pointed to the miRNAs as putatively responsible for the underlying mechanisms connecting the SP and the female reproductive immune response, since miRNAs are well-recognized regulators of the immune system [26]. This hypothesis was confirmed in mice, showing that the SP leads to an increase of several immune-regulatory miRNAs in the endometrium, and highlighting the presence of miR-223, miR-146a and miR-155, all related to immune tolerance [17,29]. Therefore, identifying which miRNAs are involved in modulating the maternal reproductive immune environment would contribute to explaining their role in events related to endometrial receptivity and implantation [22], processes whose failure may have an immunological basis [42,43]. It has been reported, in humans and mice, that secreted endometrial miRNAs are mainly transported inside extracellular vesicles and released into the endometrial fluid, where they could act as regulators of embryo-endometrial dialog [32,33]. These findings suggest that these secreted miRNAs could also modulate the maternal intrauterine environment. To the best of our knowledge, this is the first study examining miRNA expression profiles in the culture media harvested after exposure of different segments of the sow genital tract (explants from uterus, UTJ and isthmus) to SP (from SRF, post-SRF and EE) for 16 h. Oviduct and uterine explants have been cultured from several mammalian species (including pigs), and widely used as a standardized model to evaluate in vitro female genital tract responses to different treatments, due to the fact that the epithelium is exposed, as in the in vivo situation [44,45,46,47,48]. Although this ex vivo model may not completely reflect the changes that could take place in vivo, it provides reliable and useful data that can contribute to a better understanding about the role played by SP on the secretion of miRNAs by female genital tract.

In the absence of in vitro protocols to evaluate the SP effect on sow genital tract, explants were exposed to SP (diluted 1:40 (v:v) to simulate the dilution rate of commercial AI-dose) for a 16 h period, due to the period expected for boar semen to be exposed to the lining of the genital epithelium of the sow, considering the intervals of the two AI performed per oestrus conventionally practiced elsewhere [49]. It is well known that, within a few hours after AI, an acute inflammatory response dominated by a neutrophil-influx is enhanced in the sow’s genital tract in response to spermatozoa and SP [35], decreasing at 40 h post AI [50]. In cattle, SP has been proven to modulate the immune-related gene expression in endometrial explants, in an incubation time-dependent manner [51]. In the current study, explant media were collected after 24 h of exposure to SP, since this allows evaluation of the long-lasting rather than the acute/transient inflammatory response of the female genital tract to SP, in terms of miRNAs expression. The values measured of LDH activity indicated the explants remained relatively viable throughout culture, considering that the values were similar to those reported in a previous study using cow endometrial explants [46].

The present study aimed to identify the main dysregulated miRNAs in media of uterus, UTJ and isthmus exposed to SP, and a strict filter criterion was applied (FDR < 0.05 and ≥ 2.0-FC ≤ −2.0). The first contribution of the present study was that no differentially expressed miRNAs were identified in isthmus culture medium, depicting that SP could not exert any influence on the female genital tract at that level. Our results would support the hypothesis that, under physiological conditions, SP does not reach the oviduct in pigs [10,52]. Therefore, the putative influence of SP on the female genital tract seems to be exerted, as expected, in uterus and UTJ.

Focusing on miRNA transcriptome profiling of uterine and UTJ media, 15 differentially expressed miRNAs (excluding repeated miRNAs) were identified. The results indicated at first sight that the SP mainly contributed to a global downregulation of miRNA expression in the uterus and UTJ. It is worth noting that, in contrast to the general assumption that miRNAs repress gene expression in varying degrees [53], a translational activation of target genes by miRNAs has also been reported [54]. Thus, while modulation of the genes targeted by these miRNAs is clear, their behavior is difficult to predict. Enrichment analysis evidenced that the potential target-genes of our dysregulated miRNAs were involved in several biological processes and pathways related to the immune response, such as regulation of cytokine production, cytokine–cytokine receptor interaction, or Th1 and Th2 differentiation. The main dysregulated miRNAs involved in these pathways were identified in the culture media of explants exposed to post-SRF. Accordingly, it was evidenced that the SP modulates immune-related genes’ expression in sow genital tract tissues [11], which differed depending on the SP-source used [10]. Our results suggest that SP, mainly from post-SRF, also modulates miRNAs secretion in sow genital tract tissues in vitro, which could serve as a useful complement for these previous studies, since miRNAs are ultimately responsible for the regulation of protein translation codified by these immune-related genes. Additionally, KEGG pathways analysis revealed that the predicted target-genes of our dysregulated miRNAs are related to networks that seem to be involved in the establishment of the pregnancy in the pig, mainly in events related to embryo implantation [23,24,25]. These pathways include the PI3K-Akt signaling pathway, p53 signaling pathway, cell adhesion molecules, focal adhesion, MAPK signaling pathway and Wnt signaling pathway [23,24,25]. Hence, our results suggest that SP, mainly from post-SRF, also influences miRNAs’ secretion to the extracellular environment, perhaps being involved in the immune events occurring at the peri-conception period.

One of the main findings of the current study was that the dysregulated miRNAs identified in UTJ and utero-harvested media differed depending on the SP-source (from SRF, post-SRF or EE) to which explants were exposed. In the pig, as in the human [55] and the horse [56], ejaculates are expelled in sequential fractions, the SRF and the post-SRF being the two main ejaculate-fractions [34]. The SRF is the smallest in volume (40–50 mL), holding 80–90% of the total ejaculated spermatozoa and including secretions from epididymis and prostate gland [34]. By contrast, post-SRF is the largest in volume (above 150 mL), holding 10–20% of the total ejaculated spermatozoa and containing secretions from seminal vesicles and bulbourethral glands [34]. In this context, one should note that our data match with quantitative differences in SP-proteins observed between ejaculate-fractions [57], including cytokines [58], the latter being crucial mediators of the female reproductive tract immune responses [4,8,9]. It has also been evidenced that SP-active molecules are responsible for eliciting an endometrial cytokine expression [7], modulating the maternal immune tolerance toward the conceptus [59] and conditioning the success of pregnancy. Since miRNAs have also been postulated as regulators of cytokine expression [60], it is reasonable to suggest that such expression in the female reproductive tract is modulated by the miRNAs identified in our study. Related to this, *CXCL5*, *IL1RAP* and *IL5RA* were found among the genes targeted by three dysregulated miRNAs (miR-4776-3p (down), miR-34b (down) and miR-574-5p (up), respectively) identified in uterine-media. Collectively, these results could contribute to explaining how SP-miRNAs underlie the regulation of cytokine-related gene expression in the female genital tract.

This study identified, for the first time, several dysregulated miRNAs in uterus and UTJ-culture media obtained during in vitro culture for 24 h after different SP-treatments, whose specific function, biological relevance and/or predicted target genes have not yet been reported. Specifically, regardless of the SP-source used, miR-4601, miR-2313-3p and miR6460 were downregulated in UTJ- and utero-media, and miR-4384 was downregulated in utero-media. Similarly, our results demonstrated that miR-3944-3p was downregulated in the culture medium of UTJ incubated with SP from EE. Finally, miR-1713 and miR-3104-5p were upregulated in the media of uterus incubated with SP from EE, and in that of the UTJ incubated with SP from SRF, respectively. Considering that SP modulates the female reproductive immune environment, conditioning the success of fertilization, embryo implantation and pregnancy [1,4,5], it is within the realms of possibility that some of the miRNAs outlined above could be involved in the modulation of immune-related genes expression in the female genital tract. Therefore, our results warrant further research on the specific functions of these miRNAs. 

This study also identified dysregulated miRNAs in uterus and UTJ-media whose biological relevance and predicted target genes were previously reported. miR-34b and miR-4776-3p were downregulated in utero-media, regardless of the SP-source to which the uterus was exposed. Studies performed in humans have reported that dysregulation of miR-34b expression is related to endometrial cancer and endometriosis [61,62,63], and an impaired immune function has been suggested to contribute to pathogenesis of both processes [64,65,66]. An interesting finding about miR-34b-5p is its potential involvement on embryo implantation, since a differential expression is known to occur in the endometrium of pregnant sows at the onset of pregnancy [23]. In addition, it has been reported that expression levels of *IL1RAP*, one of the miR-34b target-genes, are higher in the endometrium of pregnant than of non-pregnant sows [67]. The results of our study also indicated that this miRNA was also involved in several reproduction and immune pathways. Altogether, these findings raise the possibility that miR-34b could contribute to modulate the female reproductive immune response, playing a key role on uterine receptivity for subsequent embryo implantation. With regard to miR-4776-3p, and in spite of scarce information about its biological function, *ALOX15*, *TRIM14*, *PAK5* and *USP25*, were identified among its potential target-genes, being related to the immune response, as indicated by DAVID and KEGG databases. 

The miR-92b-5p was upregulated in the culture media of uterus’ exposed to SP from SRF and post-SRF. Salilew-Wondim et al. [68] revealed that expression levels of different miR-92 family members (including miR-92b) were downregulated in the endometrium of cows with endometritis compared with healthy counterparts, which suggests a possible involvement of this miRNA in the immune response. Related to this, the miR-17-92 miRNA cluster, which includes the miR-92 family, has been reported to be involved in regulatory T cells’ function and development [69]. In this context, one should bear in mind that these immune cells seem to play an essential role in the establishment of appropriate immune tolerance on female genital tract during early pregnancy [70], and that SP appears to modulate these T cells [71]. In addition, two studies have also reported that the expression of miR-92 family members in the endometrium of sows at day 15 is higher than in day 50 [23], and that they are upregulated in the endometrium of high-prolificacy sows at day 30–32 of gestation [72]. Collectively, these data would also suggest a key role of miR-92 family members in modulating the female reproductive tract immune response for subsequent embryo implantation in the pig. 

Our results also showed that miR-205 and miR-205a-5p were downregulated in the media of uterus’ exposed to SP from post-SRF and EE. The relevance of miR-205 on female genital tissues has been extensively reported in humans, since a dysregulation of this miRNA has been associated with endometrial cancer [73,74,75]. DAVID and KEGG databases revealed that some of miR-205 predicted target-genes, specifically *COL3A1*, *MRC1*, *PIK3CG*, *SIRT1*, *NAFTC3* and *PLCB1*, were related to the immune response. Since a close link between the immune response and cancer has also been widely described [65,66], it is plausible that these miRNAs would also be implied to modulate the immune response on endometrium. 

Finally, the results also evidenced that miR-23a-5p and miR-574-5p were respectively, down- and up-regulated in the culture media of UTJ and uterus’ incubated with SP from post-SRF. Previously, reports have shown that both miRNAs could be involved in immune functions. Specifically, it was revealed that miR-23a is involved in regulating cytokine production in macrophages [76], and in modulating immune response in sepsis [77], and miR-23a-5p in mycobacterium tuberculosis infection [78]. Similarly, miR-574-5p was dysregulated in the mediastinal lymph node in pigs infected with porcine circovirus type 2 and was predicted to be involved in the regulation of the T cell receptor signaling pathway [79]. Related to reproductive processes, a dysregulation in miR-23a and miR-574 family members has been related to pathological pregnancy [80,81] and endometrial cancer in humans [82]. Therefore, the literature indicates that these two miRNAs play a key role in immune and reproductive processes, suggesting that they could also be involved in the modulation of the female immune response. 

## 5. Conclusions

In conclusion, based on the information described above, our study is the first one to demonstrate that SP is able to elicit changes in the expression profile of miRNAs in UTJ and utero, ultimately depending on the SP-composition. Since it has been reported that SP modulates female reproductive tract immune response at the peri-conception period, this study provides new insights into the identification of known and novel miRNAs that could be responsible for modulating that process. This preliminary study could serve as a basis for further studies in order to contribute to the understanding of the role played by these miRNAs in reproductive processes. Furthermore, these results could be valuable for AI centers to improve the efficiency of AI semen dose, since they provide a new vision of the role played by the SP of the different ejaculate fractions on the reproductive tissues of the sows.

## Figures and Tables

**Table 1:** List of predicted target genes of differentially expressed miRNAs (False Discovery Rate (FDR) < 0.05 and ≥2.0-Fold Change (FC) or ≤−2.0-FC) in explant media from uterus and utero-tubal junction (UTJ) exposed to different treatments (Medium 199 (M199) supplemented with seminal plasma from different ejaculate fractions (sperm-rich fraction (SRF) and post-SRF), and from the entire recomposed ejaculate (EE)) compared with its control (C, M199 alone).

miRNA	No. of Predicted Target Genes	Names of Predicted Target Genes
miR-23a-5p	12	*GIPC3*, *ABCA1*, *MTMR4*, *VSIG1*, *GOLGA6L1*, *GOLGA6L6*, *MLIP*, *LOC100132813*, *SSMEM1*, *USH2A*, *TMEM127*, *MYEOV*
miR-34b	87	*TENM1*, *INSIG1*, *ELMOD1*, *FURIN*, *PPP6R3*, *RFX3*, *DLL1*, *RAB3C*, *ZC4H2*, *CAMSAP2*, *MAP2*, *MTF2*, *MYSM1*, *NCKAP1*, *PLEKHA1*, *SETD3*, *SLITRK3*, *STK38L*, *THRB*, *ANKS1B*, *CLINT1*, *G2E3*, *GPATCH8*, *NEUROD1*, *PIK3C2A*, *PRKAR2B*, *SOX6*, *ASCL1*, *CAMK4*, *FDX1*, *RPRD1A*, *TLNRD1*, *YTHDC2*, *YWHAG*, *APH1A*, *ARID1B*, *ATP11C*, *BRINP2*, *CBLB*, *ELMSAN1*, *ESPL1*, *F2RL2*, *GAS1*, *GBP4*, *MYF5*, *RDX*, *THAP12*, *TM9SF3*, *ATP6V0A2*, *CDK19*, *CNTNAP1*, *HOXB8*, *HOXC8*, *KCNA1*, *KIF2A*, *MARVELD2*, *MYCBP2*, *NPTN*, *QDPR*, *STK39*, *AKTIP*, *APOB*, *CTNND2*, *GABRB2*, *MTCL1*, *MYC*, *NHSL1*, *PHACTR1*, *STMN2*, *TENT4B*, *ACTL6A*, *CADM2*, *CELF2*, *FKBP1B*, *IL1RAP*, *MAPK4*, *MTDH*, *NUPL2*, *PGRMC1*, *PIEZO2*, *PTPN4*, *PTPRG*, *RALA*, *RHOH*, *TENT4A*, *WIPF3*, *XKR6*
miR-34b-3p	32	*INSIG1*, *PPP6R3*, *FURIN*, *NCKAP1*, *TENM1*, *SETD3*, *MYSM1*, *MTF2*, *SLITRK3*, *MAP2*, *CLINT1*, *PRKAR2B*, *G2E3*, *GPATCH8*, *RPRD1A*, *YWHAG*, *YTHDC2*, *CAMK4*, *TLNRD1*, *FDX1*, *F2RL2*, *BRINP2*, *ESPL1*, *GBP4*, *TM9SF3*, *KIF2A*, *NPTN*, *AKTIP*, *TENT4B*, *GABRB2*, *PGRMC1*, *NUPL2*
miR92b-5p	1	*FN3K*
miR-205	266	*RRM2B*, *MOSMO*, *MINDY2*, *CSGALNACT2*, *RAB11FIP1*, *PPP2R2D*, *DMRT1*, *CHN1*, *CDK19*, *C5orf24*, *BICC1*, *RBM47*, *PNPT1*, *PIK3CG*, *PIAS2*, *NLGN1*, *LPGAT1*, *LPCAT1*, *LIN9*, *DST*, *BTBD3*, *AAK1*, *TOPBP1*, *TAPT1*, *PTPRJ*, *PDLIM5*, *NFAT5*, *NANOS1*, *MSANTD4*, *MAP3K13*, *LRRC8B*, *LRRC19*, *ITGB8*, *FAM19A1*, *FAM122A*, *DSC2*, *CCNJ*, *C9orf153*, *AZIN1*, *ATRN*, *ZNF606*, *ZFYVE16*, *WBP2*, *UBE2R2*, *TTPAL*, *TNFAIP8*, *TMEM245*, *SSR3*, *SLC30A8*, *SFT2D1*, *RSBN1*, *RPRD1A*, *ROCK2*, *RBM4B*, *RBM39*, *PMEL*, *PLCB1*, *MRC1*, *MGRN1*, *LYPLA1*, *GALNT13*, *FAM49A*, *CPSF6*, *CMC1*, *CDH11*, *CAP2*, *CALCRL*, *CADM1*, *C11orf86*, *ATRX*, *AGFG1*, *XYLT1*, *WASHC4*, *VTI1B*, *TSPAN2*, *TRIM33*, *SLC35B3*, *SLC12A2*, *SIRT1*, *SH3BGRL2*, *RYR3*, *PRKCE*, *NUP54*, *NFATC3*, *NECAP1*, *MORC3*, *MNT*, *MELK*, *MAGI2*, *LRP6*, *LCOR*, *KIAA1841*, *HS3ST1*, *GABRG1*, *FZD3*, *ENTPD1*, *CNR1*, *CASC4*, *B4GALT6*, *ATXN1*, *ARHGAP15*, *APAF1*, *ANKRD12*, *AFTPH*, *ACTA2*, *YWHAB*, *TMEM144*, *SPANXN5*, *SLC49A4*, *SLC19A2*, *SEC62*, *RELCH*, *QKI*, *PTPRM*, *PDE3B*, *NEMP1*, *MYOC*, *MPP6*, *INO80D*, *HERC3*, *HDX*, *GEMIN2*, *EVA1C*, *ERBB4*, *DUSP7*, *DRG2*, *DLD*, *CWC27*, *CPEB2*, *CEP350*, *CDK14*, *CCDC80*, *BOLA2-SMG1P6*, *AMOT*, *ZNF644*, *UNC13C*, *TUT7*, *TOB1*, *STRBP*, *SLC15A2*, *RTN3*, *RMDN1*, *RFX3*, *RCN2*, *PLAC8*, *NOL4*, *NKD1*, *NFIB*, *LRRK2*, *HS3ST2*, *HECW1*, *HDAC9*, *GPC6*, *FOXF1*, *FAM126A*, *EZR*, *ETNK1*, *ERRFI1*, *ENAH*, *DUSP16*, *DMXL2*, *COL10A1*, *B3GNT2*, *ANKRD50*, *AFDN*, *URI1*, *TMEM132B*, *TMED4*, *TIMM8A*, *SLC35A1*, *SIPA1L1*, *SGMS1*, *SEPT4*, *SEMA3A*, *SEL1L*, *SATB2*, *RORA*, *RIPOR3*, *PMFBP1*, *PDE10A*, *P2RY1*, *MSI2*, *MARCKS*, *LZIC*, *LRP1*, *INPP4A*, *HSF5*, *HSD17B11*, *GABRA4*, *F5*, *CREB1*, *COBL*, *CLTC*, *CLDN11*, *CCDC59*, *CAP1*, *C6orf222*, *ATP8A1*, *ARMC1*, *AP1S1*, *ANKIB1*, *ADAM10*, *ZNF800*, *ZNF652*, *ZEB1*, *ZC3H12C*, *XIRP2*, *TTC33*, *TET1*, *RECK*, *RCBTB1*, *RASSF6*, *RAB21*, *PTTG1IP*, *PSD3*, *PDE1C*, *PAX9*, *NEK7*, *MIER3*, *MECP2*, *LAMC1*, *KPNA1*, *IMPAD1*, *IMPA1*, *GLRB*, *FUT9*, *ERBB3*, *ELF2*, *E2F5*, *DYNLT1*, *CSTF2*, *CREBZF*, *COL3A1*, *CCSER1*, *CARNMT1*, *C5*, *APC*, *ADAMTS9*, *ADAM7*, *ZNF655*, *ZBED4*, *YAP1*, *TP53BP2*, *TNKS*, *SUCNR1*, *SORBS1*, *SECISBP2L*, *RUVBL1*, *RBM41*, *PPM1D*, *NOTCH2*, *NACC2*, *MMP16*, *MAGI1*, *LYSMD3*, *KRTAP4-2*, *KAT2B*, *IVNS1ABP*, *FAM120B*, *EPPK1*, *COX20*, *CHCHD1*, *CCSER2*, *CAPN14*, *ALG10*, *ABCD1*
miR-574-5p	18	*CALCOCO1*, *FOXI2*, *C11orf96*, *RFX4*, *NSUN5*, *DGKG*, *TCF20*, *CCK*, *FOXN3*, *DDB1*, *DAZL*, *LARP6*, *L2HGDH*, *NRN1*, *FOXL2NB*, *MS4A7*, *IL5RA*, *HLTF*
miR-4776-3p	56	*ZNF99*, *ZNF493*, *CXCL5*, *ZNF138*, *ZNF117*, *CFL2*, *HOOK1*, *LRAT*, *ARHGAP32*, *ZNF714*, *ZNF730*, *CUL3*, *EIF5A2*, *ZNF728*, *SLC16A7*, *CADM2*, *ZNF431*, *HYPK*, *ZNF329*, *ALOX15*, *AGO3*, *GGNBP2*, *CCDC88A*, *RAB3C*, *TBCEL*, *ZNF208*, *COL11A1*, *IQSEC3*, *GLIPR1L2*, *LUZP1*, *RNF169*, *PAK5*, *PHF6*, *ZNF195*, *SDC2*, *TRIM14*, *BICC1*, *ZNF107*, *USH2A*, *ZNF468*, *GLYR1*, *MTMR9*, *YTHDF3*, *PTCHD4*, *TRIM33*, *EBF1*, *DEPDC4*, *ZNF737*, *ESYT3*, *TAX1BP1*, *ZNF732*, *ZNF708*, *ZNF600*, *USP25*, *RP2*, *FBXO22*
miR-3944-3p	1	*ICAM2*

**Table 2:** The main immune- and reproduction-related pathways examined with the KEGG (Kyoto Encyclopedia of Genes and Genomes) database for predicted target-genes of dysregulated miRNAs (False Discovery Rate < 0.05 and ≥2.0-Fold Change or ≤−2.0) in the supernatants of genital sow explants (uterus and/or utero-tubal junction) exposed to Medium 199 (M199) supplemented with seminal plasma (SP) from different ejaculate fractions (sperm-rich fraction (SRF) and post-SRF) and from the recomposed ejaculate (EE) compared with its control (M199 alone). The dysregulated miRNAs were: miR-34b, miR-92b-5p, miR-34b-3p and miR-3944-3p in SRF; miR-23a-5p, miR-34b, miR-92b-5p, miR-205, miR-574-5p and miR-4776-3p in post-SRF; and miR-34b, miR-205, miR-3944-3p and miR-4776-3p in EE.

Pathway	SP-Source			miRNAs
	SRF	Post-SRF	EE	
Th1 and Th2 cell differentiation	+	+	+	miR-34b, miR-205
Cytokine-cytokine receptor interaction	+	+	+	miR-34b, miR-574-5p
T cell receptor signaling	+	+	+	miR-34b, miR-205, miR-4776-3p
IL-17 signaling	+	+	+	miR-34b, miR-4776-3p
MAPK signaling	+	+	+	miR-34b, miR-205
Chemokine signaling	−	+	+	miR-205
Th17 cell differentiation	+	+	+	miR-34b, miR-205
TGF-beta signaling	+	+	+	miR-34b, miR-205
PI3K-Akt signaling	+	+	+	miR-34b, miR-34b-3p, miR-205
Cell adhesion molecules	+	+	+	miR-34b, miR-205, miR-4776-3p
TNF signaling	−	+	+	miR-205
Wnt signaling	+	+	+	miR-34b, miR-205
Focal adhesion	+	+	+	miR-205, miR-4776-3p
GnRH signaling	−	+	+	miR-205
B cell receptor signaling	−	+	+	miR-205
Oxytocin signaling	+	+	+	miR-34b, miR-34b-3p, miR-205
JAK-STAT signaling	+	+	+	miR-34b, miR-205, miR-574-5p
Estrogen signaling	−	+	+	miR-205
p53 signaling	−	+	+	miR-205

## Supplementary Materials

The following are available online at https://www.mdpi.com/2218-273X/10/6/933/s1, Figure S1: Principal Component Analysis (PCA) analysis of microRNAs (miRNAs) expression data from media of cultured explants (uterus, utero-tubal junction (UTJ) and isthmus) incubated in Medium 199 from three sows. The green, red, blue and points represent uterine, UTJ and isthmic culture media of each sow (*n* = 3), Figure S2: Venn diagram depicting the microRNAs (miRNAs) differentially regulated (*p*-value < 0.05 and ≥2.0-Fold Change or ≤−2.0) and overlapped in media of each cultured explant (uterus, utero-tubal junction (UTJ) and isthmus) exposed to different treatment (Medium 199 (M199) supplemented with seminal plasma from different ejaculate fractions (sperm-rich fraction (SRF) and post-SRF) and the recomposed ejaculate (EE) compared with its control (C, M199 alone). (A) MiRNAs downregulated (left) and upregulated (right) in uterine-medium. (B) MiRNAs downregulated (left) and upregulated (right) in UTJ-cultured medium. (C) MiRNAs downregulated (left) and upregulated (right) in the isthmic-medium, Figure S3: Venn diagram showing the microRNAs (miRNAs) differentially regulated (*p*-value < 0.05 and ≥2.0-Fold Change or ≤−2.0) in media harvested from the cultured explants and overlapped by each experimental treatment. The explants (uterus, utero-tubal junction (UTJ) and isthmus) were cultured with medium 199 (M199) alone (Control) or supplemented with seminal plasma (SP) from different ejaculate fractions (sperm-rich fraction (SRF) and post-SRF) and from the recomposed ejaculate (EE). (A) MiRNAs downregulated (up panel) and upregulated (down panel) in the explants incubated with SP from SRF. (B) MiRNAs downregulated (up panel) and upregulated (down panel) in the explants incubated with SP from post-SRF. (C) MiRNAs downregulated (up panel) and upregulated (down panel) in the explants incubated with SP from EE, Figure S4: Venn diagram depicting the microRNAs (miRNAs) differentially regulated (False Discovery Rate < 0.1 and ≥2.0-Fold Change or ≤−2.0) and overlapped in culture media harvested from each explant (uterus and utero-tubal junction (UTJ)) exposed to different treatment (Medium 199 (M199) supplemented with seminal plasma from different ejaculate fractions (sperm-rich fraction (SRF) and post-SRF) and from the entire recomposed ejaculate (EE) compared with its control (C, M199 alone). (A) MiRNAs downregulated (left) and upregulated (right) in the uterine medium. (B) MiRNAs downregulated (left) and upregulated (right) in UTJ-medium, Figure S5: Venn diagram showing the microRNAs (miRNAs) differentially regulated (False Discovery Rate < 0.1 and ≥2.0-Fold Change or ≤−2.0) in explant media and overlapped by each experimental treatment. The explants (uterus and utero-tubal junction (UTJ)) were cultured with medium 199 (M199) alone (Control) or supplemented with seminal plasma (SP) from different ejaculate fractions (sperm-rich fraction (SRF) and post-SRF) and from the entire recomposed ejaculate (EE). (A) MiRNAs downregulated in the explants incubated with SP from SRF. (B) MiRNAs upregulated in the explants incubated with SP from SRF. (C) MiRNAs downregulated in the explants incubated with SP from post-SRF. (D) MiRNAs downregulated in the explants incubated with SP from EE, Table S1: Number of differentially regulated (up/down) microRNAs (miRNAs) in explant media after each treatment compared with its respective controls (C) and applying different statistical criteria. The explants from uterus, utero-tubal junction (UTJ) and isthmus, were cultured with Medium 199 (M199) supplemented with seminal plasma from different ejaculate-fractions (the sperm-rich fraction (SRF) and the post-SRF) and the recomposed ejaculate (EE) and with M199 alone as C, Table S2: List of microRNAs (miRNAs) differentially expressed (*p*-value < 0.05 and ≥2.0-Fold Change (FC) or ≤−2.0-FC) in explant media from uterus, utero-tubal junction (UTJ) and isthmus, exposed to different treatments (Medium 199 (M199) supplemented with seminal plasma from different ejaculate fractions (sperm-rich fraction (SRF) and post-SRF) and from the recomposed ejaculate (EE) compared with its control (C, M199 alone). ID: miRNA name, Table S3: List of common microRNAs (miRNAs) differently expressed (*p*-value < 0.05 and ≥2.0-Fold Change (FC) or ≤−2.0) in culture media harvested from each explant (uterus, utero-tubal junction (UTJ) and isthmus) exposed to different treatments (Medium 199 (M199) supplemented with seminal plasma from different ejaculate fractions (sperm-rich fraction (SRF) and post-SRF) and from the recomposed ejaculate (EE) compared with its control (C, M199 alone), Table S4: List of microRNAs (miRNAs) differentially expressed (False Discovery Rate (FDR) < 0.1 and ≥2.0-Fold Change (FC) or ≤−2.0-FC) in culture media from each explant (uterus and utero-tubal junction (UTJ)) exposed to different treatments (Medium 199 (M199) supplemented with seminal plasma from different ejaculate fractions (sperm rich fraction (SRF) and post-SRF) and from the entire recomposed ejaculate (EE)),compared with its control (C, M199 alone). ID: miRNA name.
