# Supplementary material for: Seminal Plasma Modulates miRNA Expression by Sow Genital Tract Lining Explants
Source: Biomolecules. 2020 Jun 19;10(6):933. doi: 10.3390/biom10060933 (PMC7356309; doi:10.3390/biom10060933)
Supplement: Supplementary file 1 [file biomolecules-10-00933-s001.zip › Supplementary material/Supplementary Tables/Table S3.docx]

**Table S3.** List of common microRNAs (miRNAs) differently expressed (*p*-value < 0.05 and ≥2.0-Fold Change (FC) or ≤−2.0) in culture media harvested from each explant (uterus, utero-tubal junction (UTJ) and isthmus) exposed to different treatments (Medium 199 (M199) supplemented with seminal plasma from different ejaculate fractions (sperm-rich fraction (SRF) and post-SRF) and from the recomposed ejaculate (EE) compared with its control (C, M199 alone).

| **Explant** | **Downregulated miRNAs** | **Upregulated miRNAs** |
| --- | --- | --- |
| Uterus | miR-4776-3p, miR-4601, miR-2313-3p, miR4384, **miR6460**, **miR-34b**, miR-15b-3p, **miR-34b-3p**, miR-125, miR-30c, miR-30c-5p, miR-4572, miR-29a, miR-2321, miR-99, miR-99-5p, miR-99a, miR-99a-5p, miR-4454, miR-7876-5p, miR-106b-3p, miR-27a, miR-27a-3p, miR-126, miR-126-3p, miR-126a-3p, miR-29a-3p, miR-3944-3p, miR-30a-3p, miR-30e, miR-30e-3p, miR-1271, miR-127, miR-15c-3p, miR-6987-5p, miR-20a-5p, miR-7655-3p, miR-93, miR-4472, miR-205, miR-205a-5p, miR-139, miR-139-5p, miR-181a, miR-205-5p, miR-93-5p, miR-93a, miR-106a, miR-2779, miR-106-5p, miR-106a-5p, miR-1343, miR-1343-3p | miR-92b-5p, miR-4717-3p, miR-4716, miR-4530, miR-1930-3p, **miR-4949-3p,** miR-2066, miR-2066-5p, miR-466i-5p, miR-7287-5p, miR1171, miR408a-5p, miR-I3, miR-5739, miR-1713, miR1150.2 |
| UTJ | miR-4601, miR-4776-3p, **miR6460**, miR4384, miR-2313-3p, **miR-34b**, **miR-34b-3p**, miR-15b-3p, miR-4572, miR319a-3p, miR-6977-5p, miR-3944-3p, miR-7641, miR-15c-3p, let-7c, miR-2321, miR-127, miR-127-3p, miR-1382, miR-3049-3p, miR-5100, miR-6871-5p, miR-H16-5p, miR-3147, miR-243-3p, miR-4454, miR-US4-5p, miR-885 | miR-7475-5p, miR-92b-5p, miR6446, miR-1548-5p, **miR-4949-3p**, miR-5739, miR-2066, miR-2066-5p, miR-3175, miR-936, miR-184-5p, miR-1713 |
| Isthmus | miR-3049-3p, miR161-3p.2, miR-1-5p, **miR-34b-3p**, miR-2d, **miR6460**, **miR-34b**, miR-680, miR-m01-4-5p, miR397b-3p | **miR-4949-3p**, miR-BART16, miR-3075, miR-468-3p, miR-669n |

*Common down or upregulated miRNAs among the three explants-supernatants are highlighted in boldface type
