# Supplementary material for: Seminal Plasma Modulates miRNA Expression by Sow Genital Tract Lining Explants
Source: Biomolecules. 2020 Jun 19;10(6):933. doi: 10.3390/biom10060933 (PMC7356309; doi:10.3390/biom10060933)
Supplement: Supplementary file 1 [file biomolecules-10-00933-s001.zip › Supplementary material/Supplementary Tables/Table S1.docx]

**Table S1.** Number of differentially regulated (up/down) microRNAs (miRNAs) in explant media after each treatment compared with its respective controls (C) and applying different statistical criteria. The explants from uterus, utero-tubal junction (UTJ) and isthmus, were cultured with Medium 199 (M199) supplemented with seminal plasma from different ejaculate-fractions (the sperm-rich fraction (SRF) and the post-SRF) and the recomposed ejaculate (EE) and with M199 alone as C.

| **Comparison** | **Up- or down-regulated** | **Explant** | **No. of regulated miRNAs** | | |
| --- | --- | --- | --- | --- | --- |
|  |  |  | **P-*value* ˂ 0.05 and ≥ 2.0-FC or ≤ -2.0-FC** | **FDR < 0.1 and ≥ 2.0-FC or ≤ -2.0-FC** | **FDR < 0.05 and ≥ 2.0-FC or ≤ -2.0-FC** |
| SRF *vs* C | Down | Uterus | 83 | 6 | 6 |
|  |  | UTJ | 73 | 5 | 5 |
|  |  | Isthmus | 76 | 0 | 0 |
|  | Up | Uterus | 75 | 2 | 1 |
|  |  | UTJ | 54 | 2 | 1 |
|  |  | Isthmus | 29 | 0 | 0 |
| POST-SRF *vs* C | Down | Uterus | 143 | 8 | 8 |
|  |  | UTJ | 91 | 7 | 4 |
|  |  | Isthmus | 127 | 0 | 0 |
|  | Up | Uterus | 54 | 3 | 2 |
|  |  | UTJ | 36 | 0 | 0 |
|  |  | Isthmus | 25 | 0 | 0 |
| EE *vs* C | Down | Uterus | 161 | 9 | 8 |
|  |  | UTJ | 134 | 11 | 5 |
|  |  | Isthmus | 31 | 0 | 0 |
|  | Up | Uterus | 62 | 2 | 1 |
|  |  | UTJ | 34 | 0 | 0 |
|  |  | Isthmus | 14 | 0 | 0 |

Fold Change (FC); False Discovery Rate (FDR)
