# Supplementary figures and images for: Seminal Plasma Modulates miRNA Expression by Sow Genital Tract Lining Explants
Source: Biomolecules. 2020 Jun 19;10(6):933. doi: 10.3390/biom10060933 (PMC7356309; doi:10.3390/biom10060933)

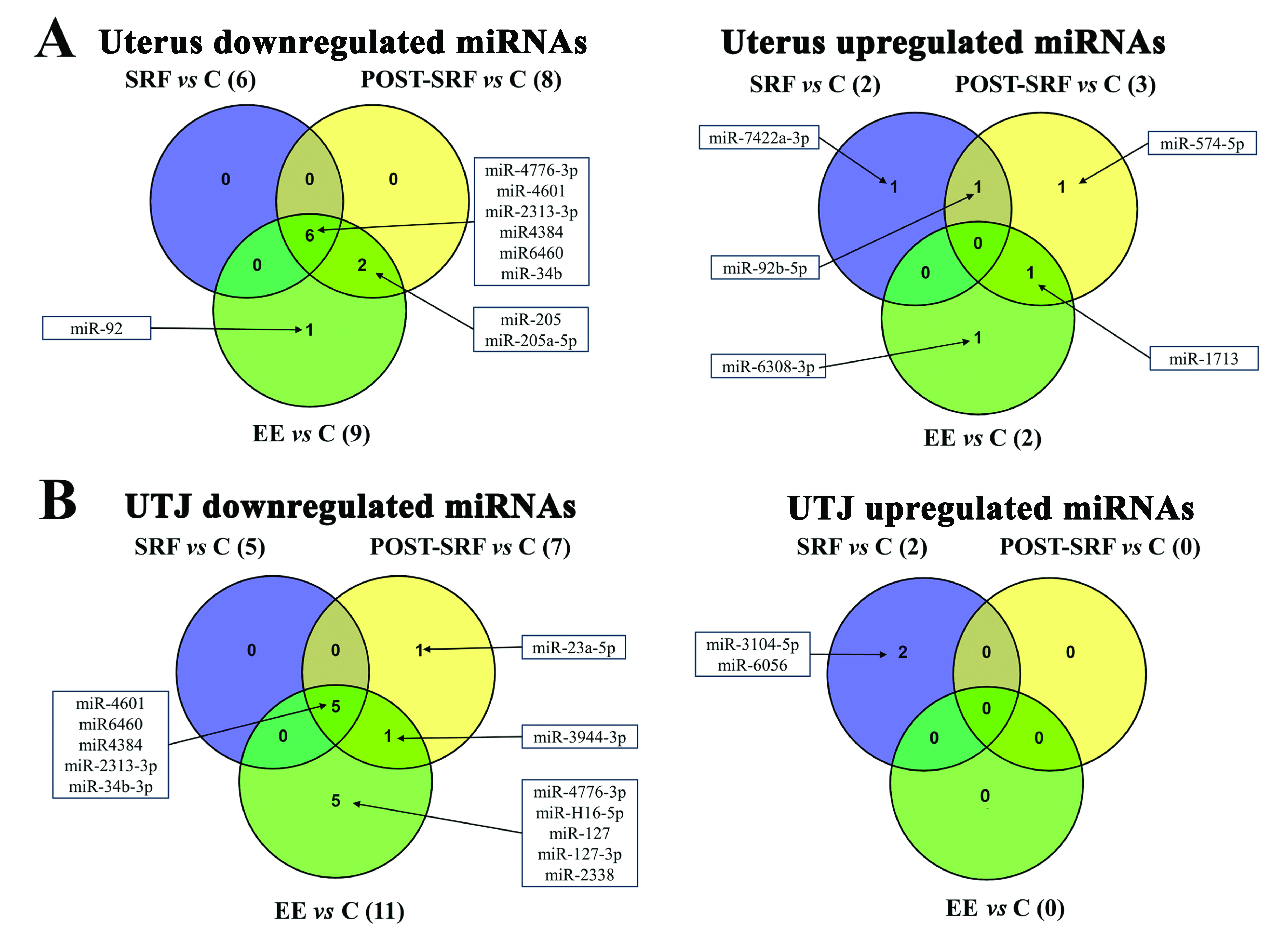

Supplement: Supplementary file 1 [file biomolecules-10-00933-s001.zip › Supplementary material/Supplementary Figures/Figure S4.tif]

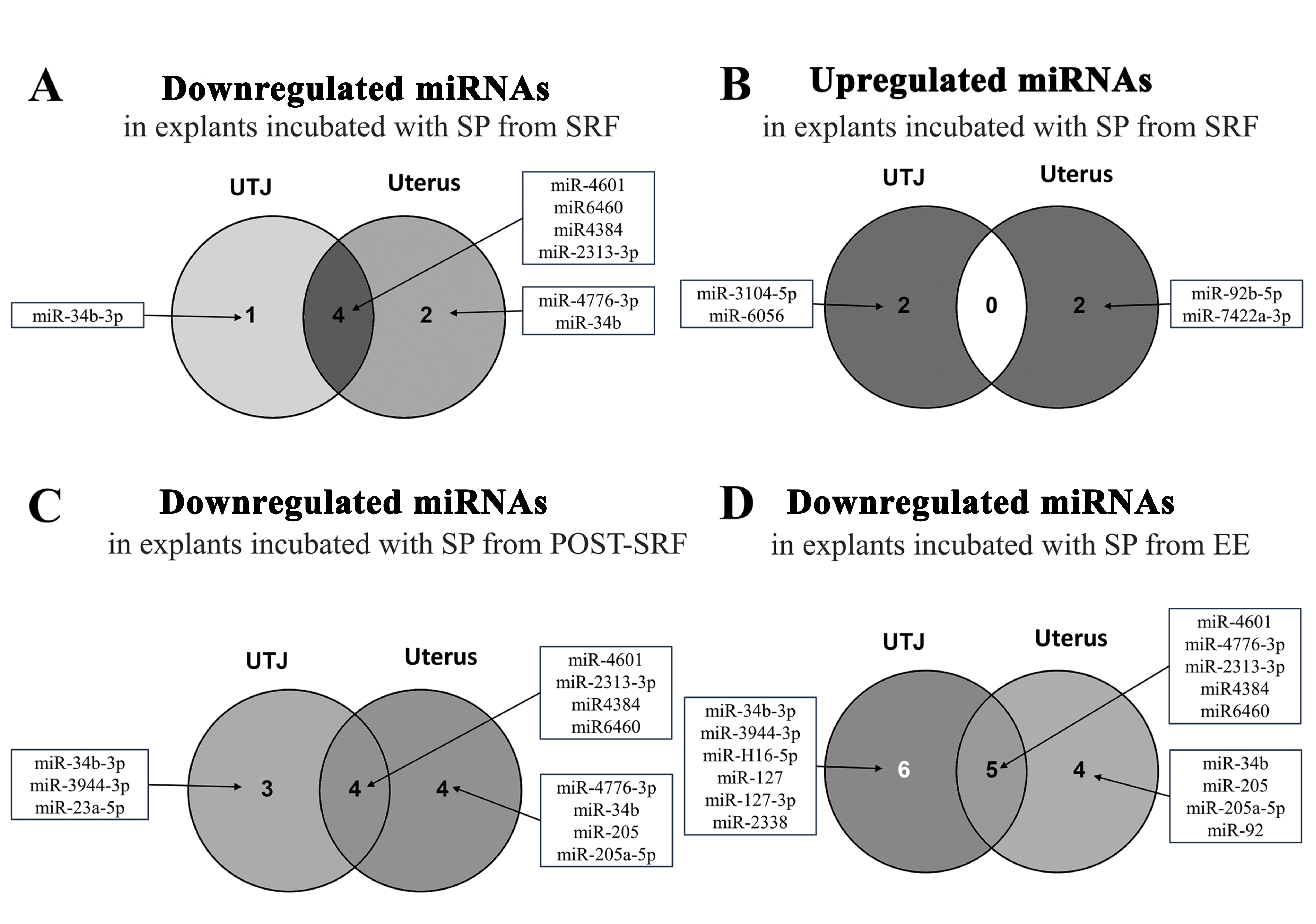

Supplement: Supplementary file 1 [file biomolecules-10-00933-s001.zip › Supplementary material/Supplementary Figures/Figure S5.tif]

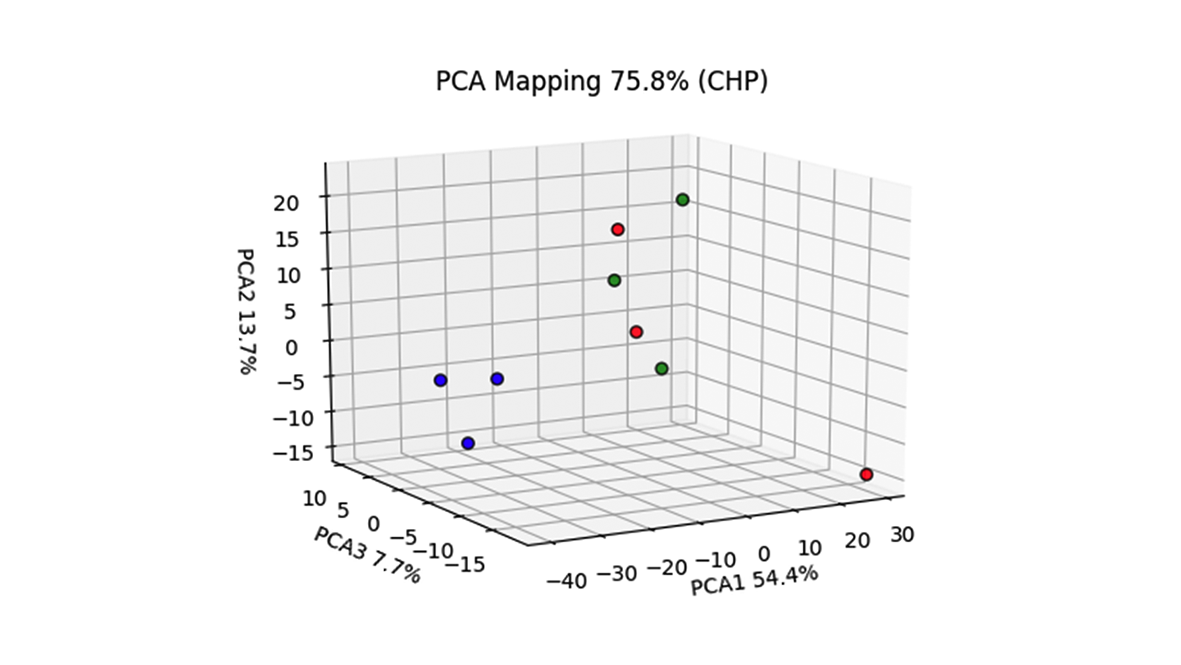

Supplement: Supplementary file 1 [file biomolecules-10-00933-s001.zip › Supplementary material/Supplementary Figures/Figure S1.tif]
